# Insulin Crystals Grown in Short-Peptide Supramolecular Hydrogels Show Enhanced Thermal Stability and Slower Release Profile

**DOI:** 10.1021/acsami.1c00639

**Published:** 2021-03-04

**Authors:** Rafael Contreras-Montoya, María Arredondo-Amador, Guillermo Escolano-Casado, Mari C. Mañas-Torres, Mercedes González, Mayte Conejero-Muriel, Vaibhav Bhatia, Juan J. Díaz-Mochón, Olga Martínez-Augustin, Fermín Sánchez de Medina, Modesto T. Lopez-Lopez, Francisco Conejero-Lara, José A. Gavira, Luis Álvarez de Cienfuegos

**Affiliations:** † Departamento de Química Orgánica, Universidad de Granada, (UGR), C. U. Fuentenueva, Avda. Severo Ochoa s/n, E-18071 Granada, Spain; ‡ Instituto de Investigación Biosanitaria ibs.GRANADA, 18014 Granada, Spain; § Laboratorio de Estudios Cristalográficos, Instituto Andaluz de Ciencias de la Tierra (Consejo Superior de Investigaciones Científicas-UGR), Avenida de las Palmeras 4, Armilla, 18100 Granada, Spain; ∥ Departamento de Farmacología, Centro de Investigación Biomédica en Red de Enfermedades Hepáticas y Digestivas (CIBERehd), School of Pharmacy, Instituto de Investigación Biosanitaria ibs.GRANADA, University of Granada, 18071 Granada, Spain; ⊥ Lamark Biotech Pvt. Ltd., VIT-TBI, 632 014 Vellore, Tamil Nadu, India; # Departamento de Química Farmacéutica y Orgánica, Facultad de Farmacia, UGR, 18011 Granada, Spain; ∇ Centre for Genomics and Oncological Research, Pfizer/University of Granada/Andalusian Regional Government, PTS Granada, Avenida de la Ilustración 114, 18016 Granada, Spain; ○ Departamento de Bioquímica y Biología Molecular II, Centro de Investigación Biomédica en Red de Enfermedades Hepáticas y Digestivas (CIBERehd), School of Pharmacy, Instituto de Investigación Biosanitaria ibs.GRANADA, University of Granada, 18071 Granada, Spain; ◆ Departamento de Física Aplicada, Facultad de Ciencias, UGR, C. U. Fuentenueva, Avda. Severo Ochoa s/n, E-18071 Granada, Spain; ¶ Instituto de Investigación Biosanitaria ibs.GRANADA, 18014 Granada, Spain; †† Departamento de Química Física, Facultad de Ciencias, UGR, C. U. Fuentenueva, Avda. Severo Ochoa s/n, E-18071 Granada, Spain

**Keywords:** insulin composite crystals, protein therapeutics, drug delivery, protein crystallization, supramolecular hydrogels, composite materials

## Abstract

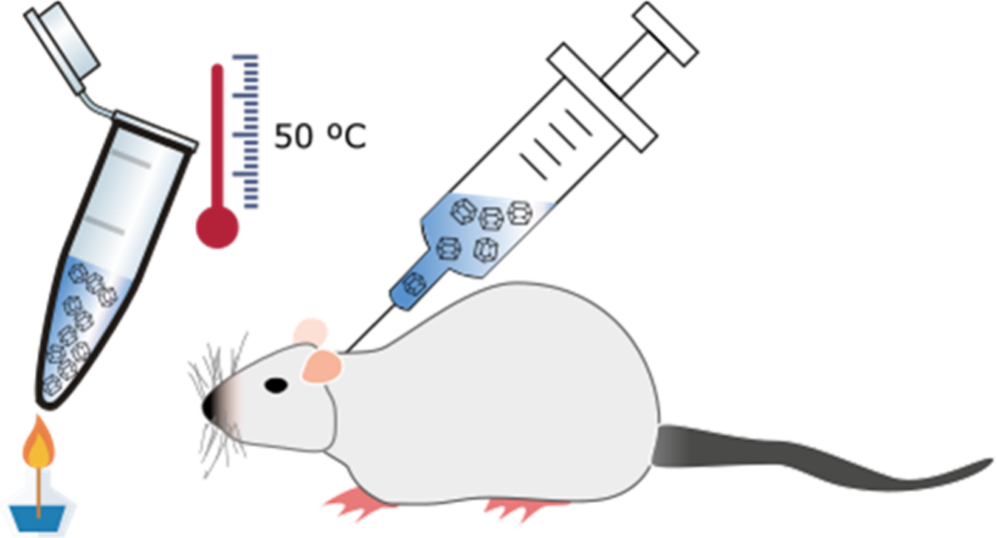

Protein therapeutics have a major role in medicine in that they are used to treat diverse pathologies. Their three-dimensional structures not only offer higher specificity and lower toxicity than small organic compounds but also make them less stable, limiting their *in vivo* half-life. Protein analogues obtained by recombinant DNA technology or by chemical modification and/or the use of drug delivery vehicles has been adopted to improve or modulate the *in vivo* pharmacological activity of proteins. Nevertheless, strategies to improve the shelf-life of protein pharmaceuticals have been less explored, which has challenged the preservation of their activity. Herein, we present a methodology that simultaneously increases the stability of proteins and modulates the release profile, and implement it with human insulin as a proof of concept. Two novel thermally stable insulin composite crystal formulations intended for the therapeutic treatment of diabetes are reported. These composite crystals have been obtained by crystallizing insulin in agarose and fluorenylmethoxycarbonyl-dialanine (Fmoc-AA) hydrogels. This process affords composite crystals, in which hydrogel fibers are occluded. The insulin in both crystalline formulations remains unaltered at 50 °C for 7 days. Differential scanning calorimetry, high-performance liquid chromatography, mass spectrometry, and *in vivo* studies have shown that insulin does not degrade after the heat treatment. The nature of the hydrogel modifies the physicochemical properties of the crystals. Crystals grown in Fmoc-AA hydrogel are more stable and have a slower dissolution rate than crystals grown in agarose. This methodology paves the way for the development of more stable protein pharmaceuticals overcoming some of the existing limitations.

## Introduction

Thanks to the advance of the recombinant DNA technology, the number of therapeutic proteins that are used for the treatment of different diseases has increased enormously in recent years, revolutionizing the pharmaceutical industry.^1^ The use of proteins for therapeutic purposes has a series of advantages in terms of specificity and safety when compared with small synthetic molecules. However, the complex structure of proteins makes them very challenging to both stabilize and preserve before administration, which translates into lower bioavailability and half-life once administered *in vivo*, limiting their therapeutic effect.^2^ Strategies developed to extend protein *in vivo* half-life include protein engineering and/or chemical post-modification with polyethylene glycol, fatty acids, and polysaccharides to name just a few.^3^ Although these techniques have proven effective, their success cannot be predicted and, in some cases, the modification can either reduce the basal activity of the protein or even produce adverse effects. Thus, alternative strategies that do not modify the chemical composition and tridimensional structure of the proteins have been explored.^4^ Moreover, efforts have been devoted to develop systems that can exert a controlled drug release.^5^

Among the different delivery systems, microparticles, emulsions, etc., hydrogels seem to be ideal materials as they are biocompatible and biodegradable and, therefore, can be used for *in vivo* applications and can also be prepared under mild conditions, thus preserving the protein stability.^6^ Hydrogels have been intensively studied as protein carriers that can modulate the protein release profile based on the chemical nature of the hydrogel, concentration, and type and degree of crosslinking.^7,8^ In particular, injectable hydrogels have attracted a great interest due to the capacity of *in vivo* administration by noninvasive injection methods.^9,10^ Nevertheless, most of these hydrogel systems have exclusively focused on controlling the release rate of the protein and less attention has been paid to developing hydrogels that can improve the stability of the protein, although this is the center of active research.^11,12^ The preservation of a protein’s native structure is one of the main issues for many hydrogel formulations that limits their application in clinics.^6^ Besides, proteins in crystalline form can show some advantages when intended for therapeutic uses, such as ease of handling, higher concentration doses per volume than their soluble format, varied dissolution rates, and even improved stability.^13,14^ In this respect, herein we have developed a strategy to create novel protein delivery formulations that can simultaneously improve protein stability and modify the release profile. This strategy uses hydrogels as media for protein crystallization. When protein crystals are grown in hydrogel media, the hydrogel material is occluded inside the crystals, giving rise to composite protein crystals.^15^ We have proven that owing to its inherent nature, the hydrogel is able to modify certain characteristics of the crystals, such as quality, polymorphism, etc.^16−22^ Herein, we show that the hydrogel can modify the *in vitro* and *in vivo* release profiles of insulin crystals and, at the same time, enhance the stability of the protein in its crystal form. In particular, short-peptide supramolecular hydrogels^23,24^ are able to stabilize insulin crystals to a higher degree, slowing down their release, compared to agarose crystals and the crystal control grown without hydrogel.

## Results and Discussion

**Figure 1:**
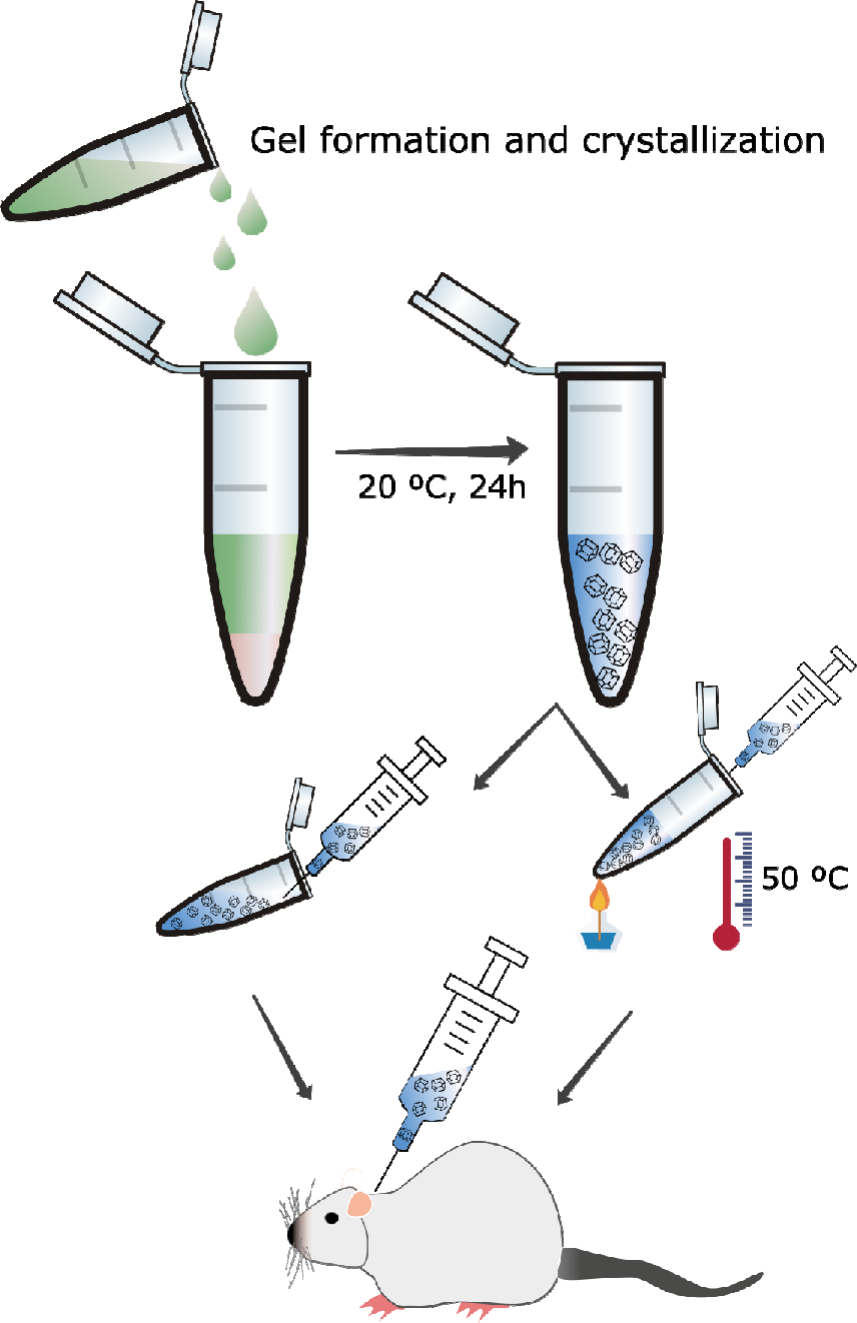
Schematic chart of the preparation of composite insulin crystal formulation for *in vivo* administration.

First, we developed a novel protocol to produce homogeneous batches of small crystals with a very narrow size distribution (4 ± 1 μm) inside injectable and biocompatible hydrogels that allowed direct subcutaneous administration (Figure 1). To do so, the selection of the hydrogel was crucial since the hydrogel is responsible for (1) controlling the quality, size, and homogeneous distribution of the crystals, i.e., dissolution rates; (2) conferring improved stability to the crystals against mechanical stress and temperature; and (3) being a biocompatible and injectable matrix that can be easily reconstituted to allow homogeneous resuspension of the crystals for adequate administration. Based on our previous studies, we selected agarose, fluorenylmethoxycarbonyl-dialanine (Fmoc-AA), and Fmoc-diphenylalanine (Fmoc-FF) hydrogels since these are physical hydrogels that at lower concentrations are weak, can be taken by a syringe, and, as we have previously shown, are compatible with protein crystallization.^18,20^

**Figure 2:**
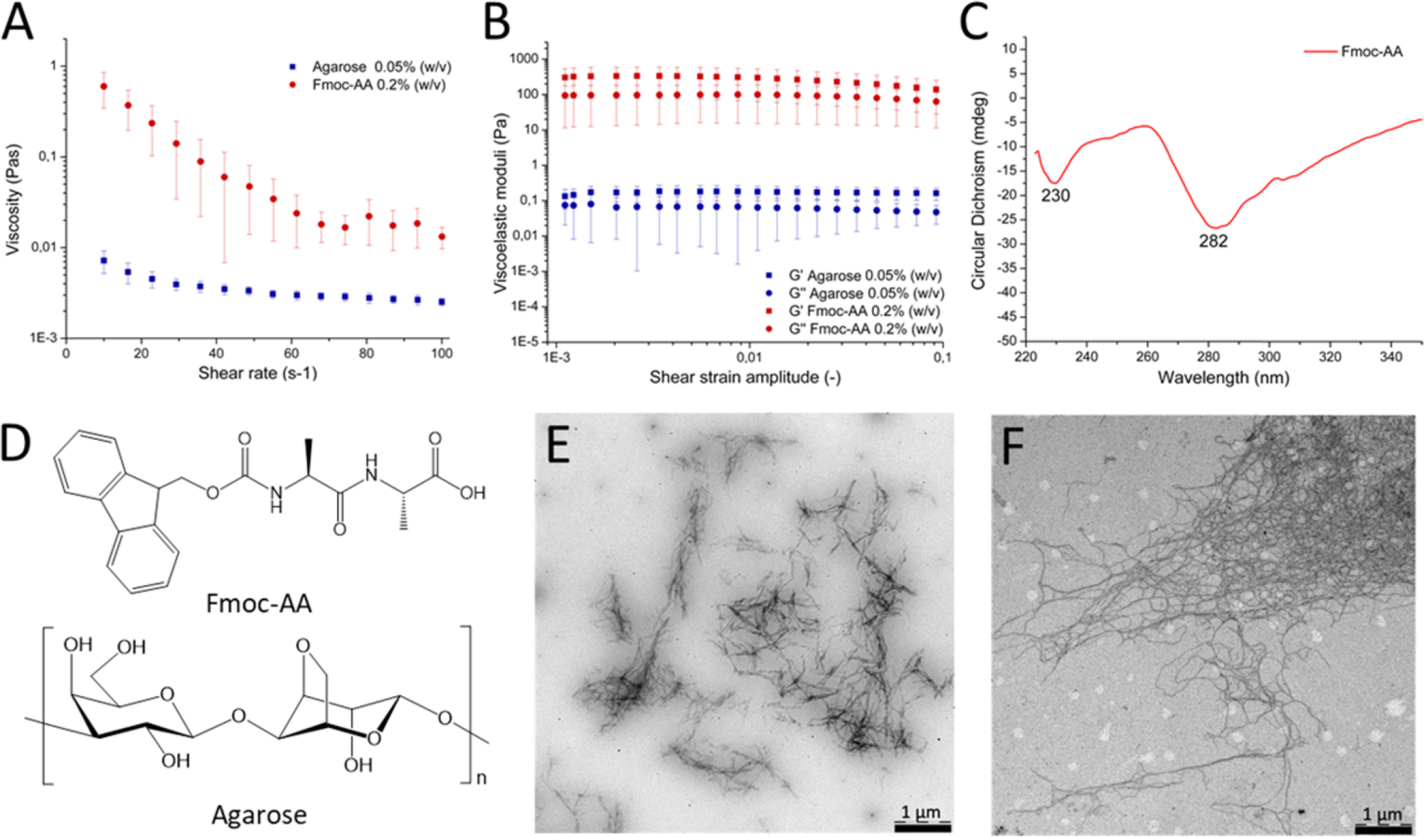
Viscosity (A) and viscoelastic moduli (B) values of the hydrogels (agarose: blue and Fmoc-AA: red). (C) circular dichroism (CD) spectra of Fmoc-AA. (D) Schematic representation of Fmoc-AA and agarose molecules. (E) Transmission electron microscopy (TEM) image of Fmoc-AA and (F) agarose dried gels.

We started by studying the compatibility of insulin with the formation of the gel as a function of the gel concentration. When Fmoc-FF was mixed with insulin, it precipitated at all concentrations tested, suggesting that a strong interaction between the peptide fiber and the protein occurred. When we carried out the same experiments with Fmoc-AA, we could avoid precipitation at a lower peptide concentration of 0.2% w/v, affording very weak hydrogels in which the protein in the solution formed the expected crystals. Agarose has been more widely studied to produce protein crystals, including insulin,^22^ and therefore we selected a low agarose concentration (0.05% w/v) to avoid any further influence on the crystallization process. Fmoc-AA hydrogels demonstrated a strong shear thinning behavior, which could be attributed to rupture of the chain-like network by the shear forces causing reduction in viscosity, confirming the gel nature of the media (Figure 2A). The low values of viscosity (for reference, the viscosity of water at 25 °C is 10^–3^ Pa·s) showed the weakness of the gel at this particular concentration, which was already observed by visual and manual exploration. To further confirm the gel-like character, we obtained the viscoelastic moduli of the sample as a function of the strain amplitude for a fixed frequency of oscillation of 1 Hz (Figure 2B). As observed, the sample showed the typical behavior of a weak gel, characterized by values of the viscoelastic moduli being approximately independent of the strain amplitude at low values of the latter (linear viscoelastic region), with the storage modulus, *G*′, being higher than the loss modulus, *G*″, by less than 1 order of magnitude. At higher values of the strain amplitude, both *G*′ and *G*″ decreased rapidly (in the nonlinear viscoelastic region), and eventually, *G*″ overcame *G*′, indicating the onset of the fluid-like behavior due to the breakage of the internal structure of the gel by the shear forces.

Circular dichroism (CD) showed two distinctive negative bands at 230 and 282 nm corresponding to n−π* and π–π* transitions of the Fmoc group, in agreement with previous results reported for other Fmoc derivatives (Figure 2C). Although it is reported that these peptides preferentially arrange in β-sheets, Ren and co-workers have shown that this particular peptide adopts mainly a polyproline II conformation.^25^ These aggregates were directly observed by transmission electron microscopy (TEM) showing small fibers of approximately 20 and 500 nm of width and length, respectively (Figure 2E), a morphology frequently observed in this type of aggregates.^26^ The length of these aggregates was significantly shortened with respect to other examples at higher concentrations, which justifies the weakness of these gels.^27^ On the other hand, agarose hydrogels showed values of viscosity about 1 order of magnitude smaller than Fmoc-AA hydrogels (Figure 2A), evidencing their extremely weak nature. Nevertheless, a slight shear thinning behavior was still observed for these hydrogels, which may be interpreted as an indication of their gel nature. This gel-like character was further confirmed by the curves of *G*′ and *G*″ as a function of the shear strain amplitude, which demonstrated similar trends to those of Fmoc-AA hydrogels, although with much lower (about 3 orders of magnitude) values of the viscoelastic moduli (Figure 2B). TEM images of agarose hydrogels showed the presence of dense meshes of fiber aggregates of higher aspect ratio than those formed by Fmoc-AA (Figure 2F).

**Figure 3:**
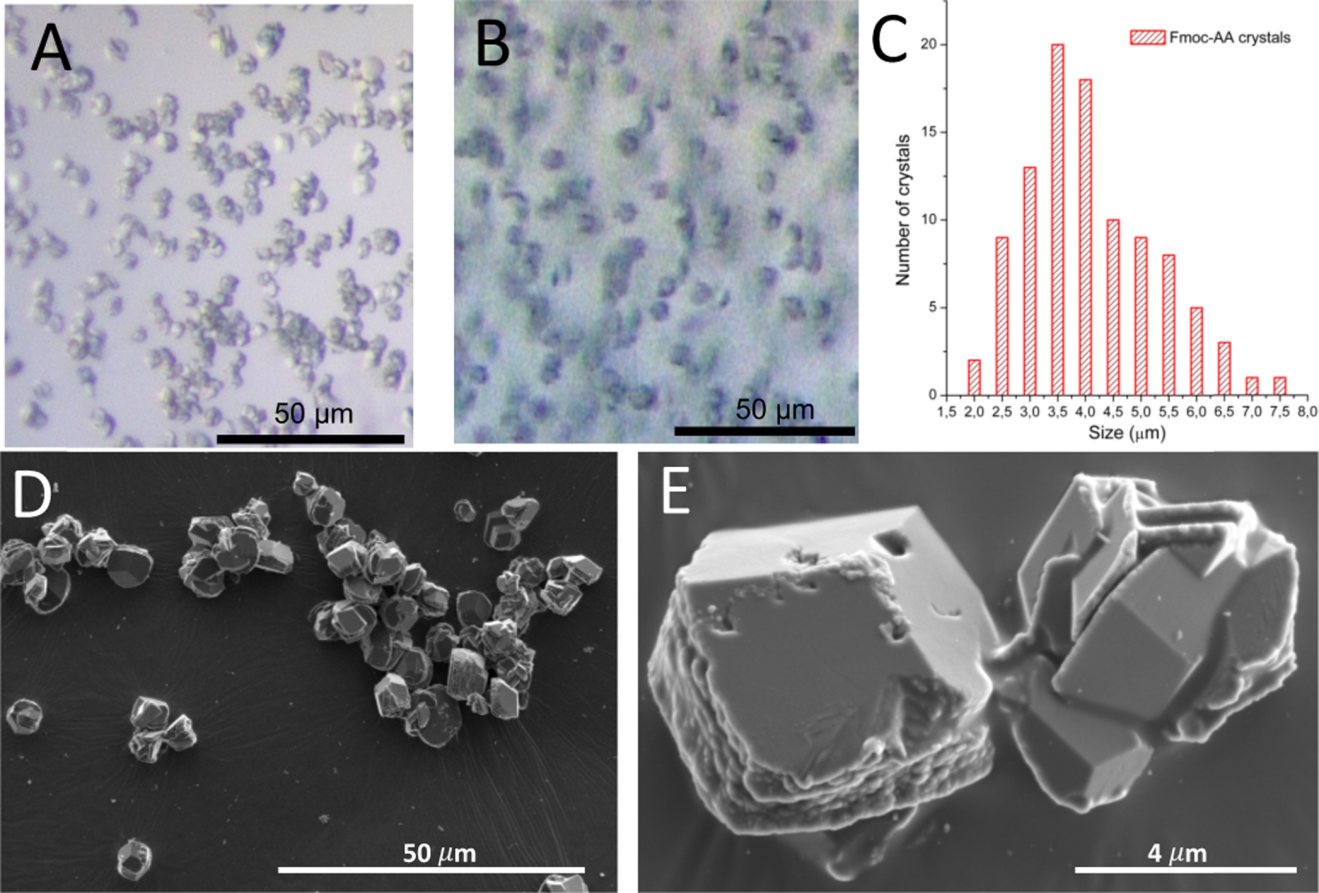
Optical microscopy images of insulin composite crystals grown in Fmoc-AA (A) and agarose (B) hydrogels. Plot of Fmoc-AA crystal size distribution (C). SEM images of Fmoc-AA crystals at 50 μm (D) and 4 μm (E) magnification.

Crystallization of insulin in agarose and Fmoc-AA hydrogels, under optimized conditions, produced homogeneous batches of small crystals (4 ± 1 μm) of very narrow size distribution (Figure 3A–C). For comparison, crystals grown without hydrogels (crystal control) were also obtained (Figure S1, Supporting Information). The size of the crystals was measured manually from scanning electron microscopy (SEM) images using only crystals with well-defined edges (Figures 3D,E, and S1 Supporting Information). As shown in Figure 3A,B, the crystals presented a rhombohedral geometry corresponding to the expected and well-described R3 space group containing two Zn atoms and six insulin molecules in the asymmetric unit, as early described by Hodgkin and co-workers in 1969.^28^ This polymorph, preferred for insulin hexamers, was expected since the crystallization was induced in the presence of Zn^2+^.^29,30^ The practical absence of morphological differences between crystals grown in hydrogels and the crystal control showed that, in this case, the hydrogel media did not influence the polymorph selection. The quality of the crystals was evaluated at a molecular level by X-ray synchrotron radiation. In spite of their small sizes, the quality of the crystals was very high, being diffracted at a resolution higher than 1.2 Å. X-ray diffraction confirmed the presence of hexameric insulin units arranged in a rhombohedral crystal polymorph. The gel fibers inside the crystals do not have a crystalline pattern and therefore cannot be detected by X-ray diffraction; neither of this contributes significantly to the X-ray background scattering.

**Figure 4:**
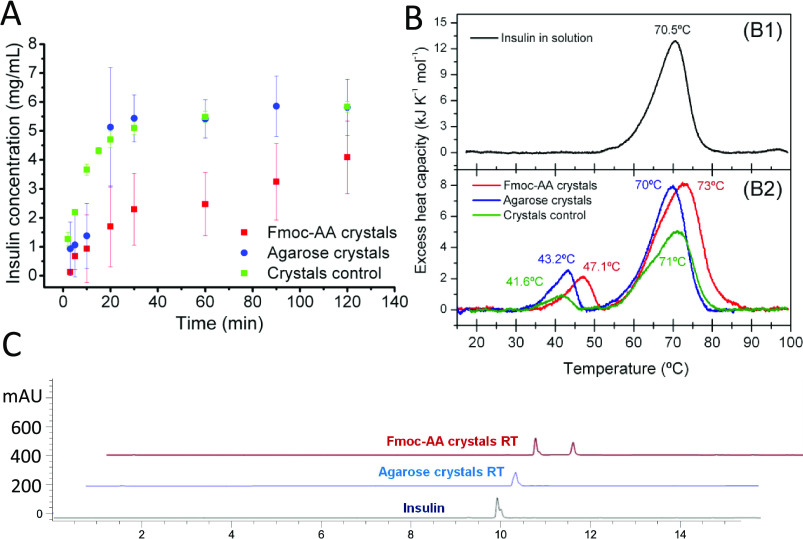
Characterization of insulin crystals maintained at room temperature. (A) Dissolution rate of insulin crystals. (B) Differential scanning calorimetry (DSC) of insulin samples. Transition temperatures are shown at the peaks. (C) High-performance liquid chromatography (HPLC) profiles of dissolved insulin crystals.

Having already obtained small insulin composite crystals of high quality, i.e., low density of crystal defects, and very narrow size distribution, we evaluated their physicochemical properties in order to study the influence of the hydrogel nature. The dissolution rate of both insulin composite crystals was measured and compared to that of crystal control under physiological conditions, that is, 38 °C and pH 7.0 (Figure 4A). To evaluate the dissolution rates of the isolated composite crystals, crystals were collected and cleaned from the hydrogel media. As we can observe from Figure 4A, dissolution rates at shorter times, i.e., below 10 min, were similar for all types of crystals; however, after that initial phase, the dissolution behavior followed different paths. Thus, control and agarose crystals showed an increased dissolution rate, levelling off at 30 min, with a typical concave curve of an inhomogeneous and accelerated dissolution rate (burst effect). In contrast, Fmoc-AA crystals presented a slower dissolution rate, levelling off at 120 min. In this case, the slope of the curve was less pronounced, showing a more linear increase of insulin concentration versus time. These results showed that the nature of the hydrogel inside the protein crystals modulates the dissolution rate. Insulin composite crystals obtained in Fmoc-AA hydrogels gave rise to crystals that had a slower dissolution rate. The reason why Fmoc-AA peptide fibers produce crystals of slower dissolution rate than agarose could be explained by the formation of stronger non-covalent interactions between the peptide fibers and the protein within the crystal. Fmoc-peptides, unlike agarose, can interact with proteins not only by the formation of hydrogen bonds but also by π–π interactions through the aromatic groups.^25^ We have recently shown that Fmoc-FF is able to efficiently interact and solvate carbon nanotubes and lysozyme crystals, proving its capacity to form strong aromatic interactions.^31^ It has also been reported that similar short peptide fibers are able to interact with proteins to different degrees.^32,33^ As previously commented, the particular conformation of polyproline II adopted by Fmoc-AA in water makes the fibers more amphiphilic, displaying on their surfaces carboxylic and Fmoc- groups and, therefore, promoting interactions with the protein through both hydrogen bonds and π–π interactions. To further investigate if this interaction occurred in solution, differential scanning calorimetry (DSC) and fluorescence measurements were performed with insulin incubated in solution with Fmoc-AA gel. In both cases, the results showed that insulin in solution did not interact with the peptide fibers, suggesting that this interaction may only occur when the crystallization conditions force the protein to nucleate and form the crystals. Under these conditions, it is known that solid particles (in this case, peptide fibers) can promote protein nucleation by acting as nucleation centers.

Next, we analyzed the resistance to thermally induced dissolution of the crystals by DSC (Figure 4B). The crystal suspensions were prediluted with crystallization buffer to 1 mg/mL of insulin concentration. The DSC thermograms of the crystals kept at room temperature (RT) showed two peaks: a small one around 40–50 °C and a bigger one at about 70 °C. Soluble insulin dissolved at 1 mg/mL in the same crystallization buffer gave rise only to the peak at 70 °C, corresponding to the thermal unfolding of insulin under the conditions of the experiments (Figure 4B). Therefore, the first small transition must correspond to the thermally induced dissolution of the insulin crystals. This peak does not occur in a second consecutive scan, as expected, since insulin does not recrystallize at 1 mg/mL under these conditions. Interestingly, crystals grown in both hydrogels were more thermally stable than the crystal control. The higher stability of Fmoc-AA crystals can be explained in the same way as the slower release profile due to stronger interactions between the protein and peptide fibers. The area under the first peak corresponds to the heat of crystal dissolution, which was similar for the two insulin composite crystals. Insulin was also more stable against thermal denaturation in the presence of Fmoc-AA, as evidenced by the higher temperature of the unfolding transition. This suggests that the interaction gain in the crystal form is somehow maintained under the experimental conditions contributing to insulin stabilization.

The integrity of insulin in the composite crystals was determined by HPLC using a diode array detector to record the UV–vis absorption spectra (Figure 4C). Samples were fractionated by HPLC using a C18 column and a gradient method. Peaks detected at 276 nm were collected and analyzed by matrix assisted laser desorption ionization-time of flight (MALDI-ToF) mass spectrometry. A calibration curve was prepared with the soluble recombinant insulin used to prepare the composite crystals by integrating the peak with a retention time of 10 min and absorbance at 276 nm (Figures S2 and S3 for crystal control, Supporting Information). A single peak at 276 nm was observed. This peak corresponded to the insulin monomer having a mass of 5808 g/mol. A Fmoc-AA gel sample prepared as for insulin crystallization was also analyzed by HPLC showing a peak absorbing at 276 nm with a retention time of 11 min. Therefore, this method was able to clearly distinguish insulin from the dipeptide. This was further demonstrated via a sample mixture analysis. Crystal samples were then dissolved, and replicates (*n* = 5) were analyzed. Sharp and time-resolved insulin peaks for all the crystal samples were obtained (Figure 4C). Integration of the peak at 10 min afforded a 5.0 ± 0.5 and 4.8 ± 0.4 mg/mL insulin concentration for agarose and Fmoc-AA crystals, respectively, in good correlation with the theoretical value (5 mg/mL). Mass analysis of these peaks corresponded to the mass of the insulin monomer, in agreement with the commercial insulin used to prepare the crystals (Figure S4, Supporting Information).

**Figure 5:**
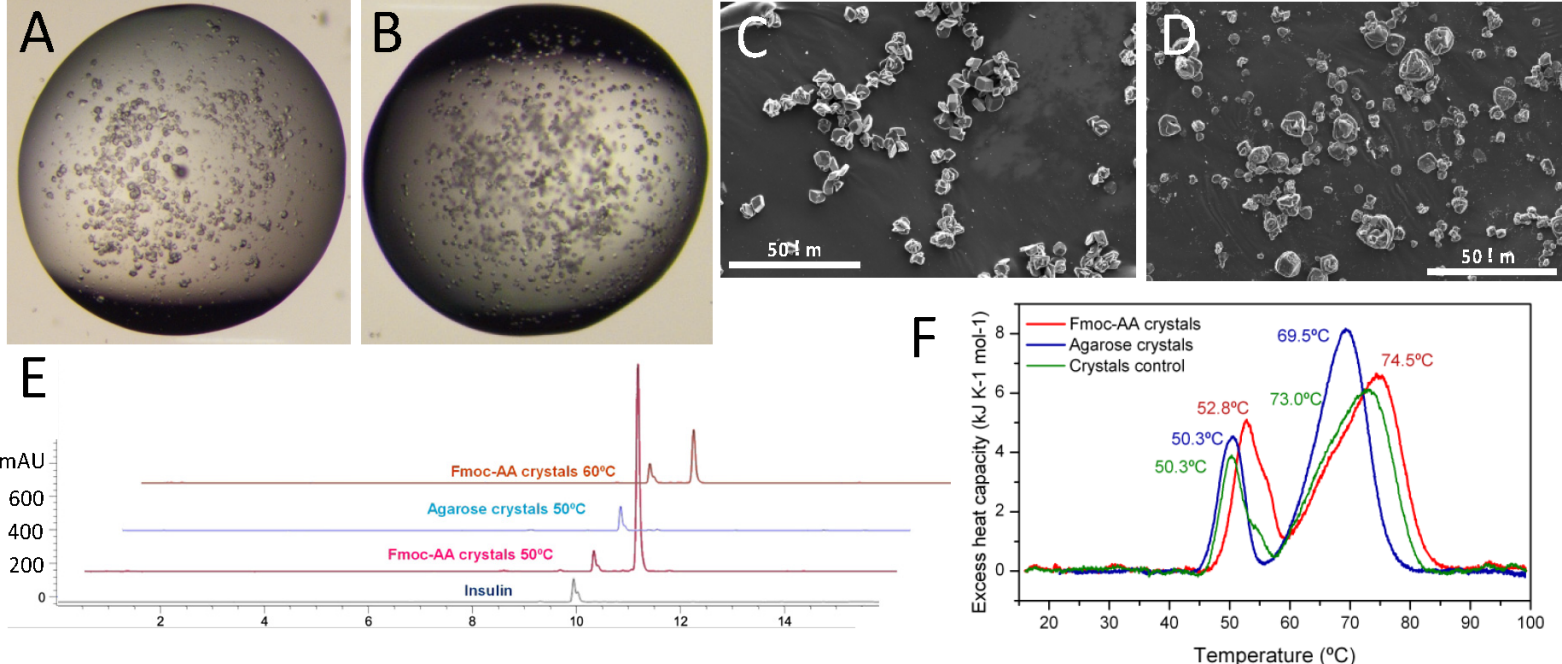
Characterization of insulin crystals kept at 50 °C for 7 days. Optical microscopy images of insulin crystals grown in Fmoc-AA (A) and agarose (B) and the corresponding SEM images of Fmoc-AA (C) and agarose (D). (E) HPLC profiles of dissolved insulin crystals. (F) Differential scanning calorimetry (DSC) of insulin samples. Transition temperatures are shown at the peaks.

Next, crystal samples were evaluated by accelerated stability studies incubating the crystal’s hydrogel suspensions at 50 °C during 7 days. It is established that incubation of crystals at 50 °C for 4 days is equivalent to a room temperature stability of 2 years.^34^ Insulin in solution degrades in less than 24 h under these conditions. Optical microscopy images were taken from day 1 to day 7 in order to evaluate the quality of the crystals and to observe differences. Fmoc-AA and agarose crystals kept at 50 °C for 7 days still showed good transparency and flat faces analogous to those kept at RT (Figure 5). Additionally, to confirm the quality of the crystals, SEM images were taken (Figure 5C,D). As can be seen from the images, the morphology, quality, and size of the crystals were similar to those samples kept at RT. We next tested the stability of the crystal suspensions at 60 °C. In this case, after 24 h, agarose and control crystals dissolved to the naked eye. Optical microscopy observation showed an amorphous precipitate in the agarose crystal sample. On the contrary, Fmoc-AA crystals were stable for up to 24 h without degradation, as monitored by optical microscopy. SEM images of the Fmoc-AA crystals kept for 24 h at 60 °C showed crystals of similar quality to those kept at RT. These results, in line with those obtained in the dissolution experiments and DSC, reveal the superior stabilizing effect exerted by Fmoc-AA peptide fibers on the crystals.

HPLC analysis of Fmoc-AA and agarose crystals kept at 50 °C and Fmoc-AA kept at 60 °C showed the same clean spectra as samples kept at RT (Figure 5E). The peak corresponding to the insulin monomer appeared again at 10 min. MALDI analysis of the peak confirmed the mass (5808 g/mol) of the insulin monomer. The practical absence of other peaks suggests that crystals do not suffer a significant degradation under these conditions.

DSC was used to evaluate the resistance against thermally induced dissolution of the crystals at 1 mg/mL kept at 50 °C for 7 days. Strikingly, the pretransitions corresponding to crystal dissolution were shifted to higher temperature for both hydrogels while their peak areas became larger (Figure 5F), indicating a considerable stabilization of the crystal structure by the heat treatment. Once more, the Fmoc-AA crystals were more stable than the agarose crystals but both became stabilized by the heat treatment to a similar extent. This increase in stability suggests that the interactions between proteins and protein-gel fibers within the crystal increased or became stronger after the heat treatment. Nevertheless, the clean HPLC and mass spectra of these samples show that this change is reversible and therefore the increase in stability must be due to the noncovalent interactions.

**Figure 6:**
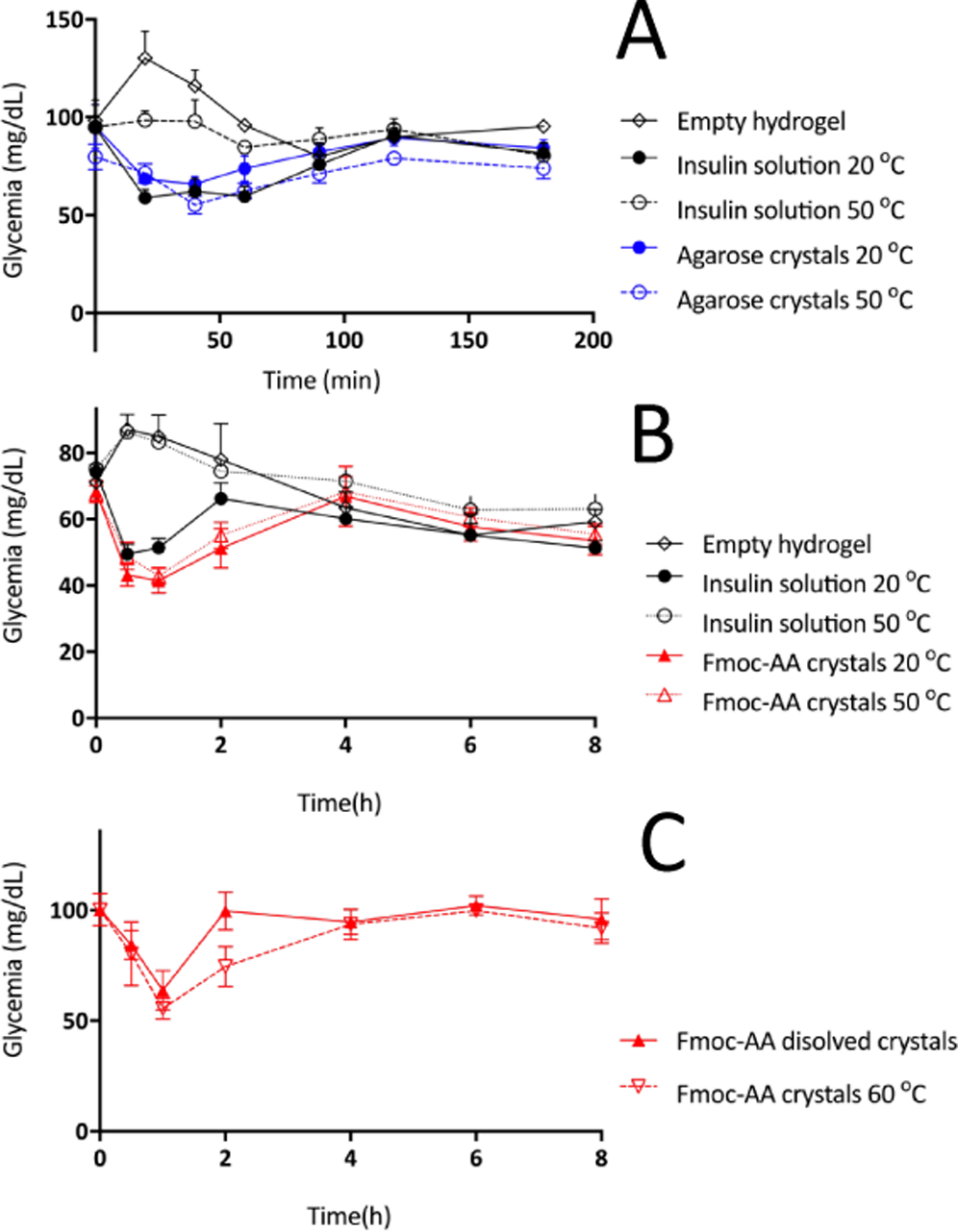
(A) Glycemia profile of agarose crystals at 20 and 50 °C. (B) Glycemia profile of Fmoc-AA crystals at 20 and 50 °C. (C) Glycemia profile of dissolved Fmoc-AA crystals kept at 50 °C and Fmoc-AA crystals kept at 60 °C. Reference insulin (black), agarose crystals (blue), and Fmoc-AA crystals (red).

The pharmacological effect of the novel insulin composite crystals was evaluated *in vivo* including samples kept at 50 and 60 °C. Native insulin in soluble form was used as a reference. Human insulin shows bioactivity in rodents and, as in humans, hypoglycemic activity is related to the concentration of insulin monomers in blood. Since the insulin molecule was the same in all preparations, the hypoglycemic response is directly related to the rate of insulin release from the injection site. In this regard, our assay is pharmacokinetic in nature. First, we conducted a dose-finding study for reference insulin, on the basis of published studies,^35−38^ in order to establish a dose associated with a robust, nontoxic, and reproducible decrease in the glucose plasma level in rats (data not shown). The dose selected, 16.7 μg/kg rat (or 435 mIU/kg), produced reliably a 30–40% reduction of glycemia in these conditions (Figure 6). As expected, glucose lowering started 20–30 min after administration in all cases and lasted for a little over an hour. The vehicle groups exhibited a trend toward an increase immediately after administration, which probably reflects a physiological response to the injection, as expected in conscious animals, and is blunted in the insulin-treated groups. As shown in Figure 6A, a similar hypoglycemic response was attained when the same dose of insulin was administered in the form of agarose crystals, with no discernible differences in the starting time or overall duration. Insulin control showed the same release profile as agarose crystals. In contrast, no response was observed in the case of Fmoc-AA crystals (not shown). As this lack of biological effect suggested a markedly slower rate of release to the bloodstream, larger doses, similar to those use for slow-release insulin glargine, were tested.^39^ In order to obtain an effect comparable to that of reference insulin, the dose had to be increased 5-fold (i.e., 83.7 μg/kg), as shown in Figure 6B. The effect took slightly longer to set in; therefore, the sample collection pattern was changed to adapt to the different response and also to assess the possibility of a longer duration of that effect. Note that neither the empty hydrogels nor the insulin composite crystals produce any observable adverse effect in the animal during the whole treatment.

The resistance to heat of insulin crystals grown in both hydrogels was assessed by incubation of the crystals at 50 °C during 7 days (Figure 6A,B) and, in the case of Fmoc-AA crystals, also at 60 °C for 24 h (Figure 6C). The reference was clearly shown to lose biological activity in these conditions. In turn, insulin crystals grown in both hydrogels remained fully active. Our data indicate that (1) the agarose hydrogel does not affect insulin release from the injection site; (2) the Fmoc-AA hydrogel has, on the contrary, a marked slowing effect on this critical pharmacokinetic step; (3) both hydrogels fully protect insulin against the thermal challenge; and (4) neither agarose nor Fmoc-AA lowers glycemia by itself. Thus, in both instances, hydrogels may be useful to improve the stability of insulin and potentially other therapeutic proteins. As a rule, therapeutic proteins must be kept from high temperatures and handled adequately to avoid deterioration, which not only may reduce the therapeutic effect (as was the case here with reference insulin) but also may lead to enhanced immunogenicity.^14,34^

On the other hand, it is interesting to consider the different impacts of the hydrogels on the insulin monomer release, as assessed by the resulting hypoglycemic response. Fmoc-AA had a major effect at this level, as opposed to agarose, which was neutral. The slower release profile *in vivo* of insulin grown in Fmoc-AA hydrogels can be correlated with the slower dissolution rate in near-physiological conditions and can be justified, as commented earlier, by the stabilizing effect exerted on the crystals by this peptide. This effect could be compensated by increasing the insulin dose. With 5-fold higher levels than initially tested, the effect was comparable to the reference insulin in solution, although the effect onset tended to be somewhat slower. Even so, the duration of the effect was never consistently higher, even with higher concentrations/doses. The reason for this observation is still unclear, but it is likely that part of the insulin–gel composites release very slowly after the first 3–4 h. Such minimal input to the bloodstream insulin thus may be matched by hepatic catabolism, resulting in a lack of effect. This would explain the results obtained with the initial 16.7 μg/kg dose. To demonstrate the slow-release effect exerted by Fmoc-AA peptide over the crystals and the preservation of the biological activity of the crystals, we tested a soluble insulin formulation prepared by dissolving Fmoc-AA crystals that had been kept at 50 °C during 7 days. As can be seen in Figure 6C, the 1-fold injection produced a similar pharmacological effect in terms of hypoglycemic response and onset time as that for native insulin in soluble form used as a reference. This result showed that Fmoc-AA modifies the rate of release of insulin into the bloodstream without altering its pharmacological activity.

**Figure 7:**
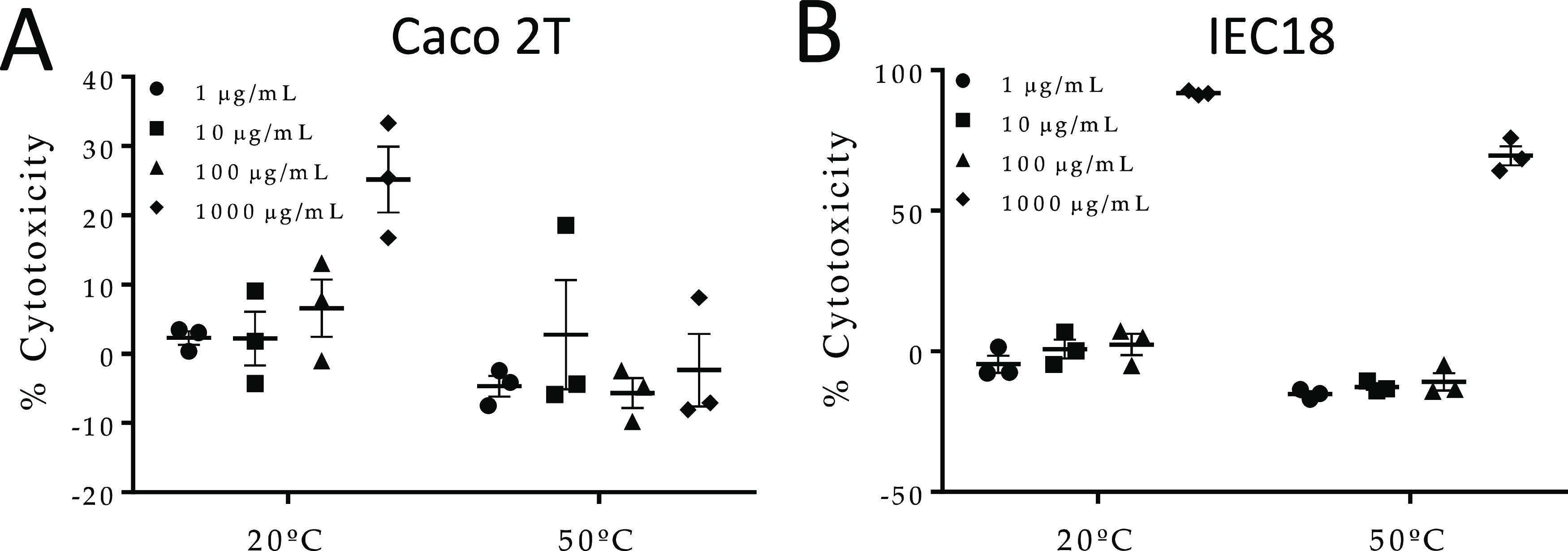
*In vitro* cytotoxic assay: Caco 2T (A) and IEC18 (B). Cells were submitted to increasing concentrations of Fmoc-AA hydrogel (1–1000 μg/mL) kept at 20 or 50 °C for 7 days. Toxicity was measured by LDH cytotoxicity assay following the manufacturer’s instructions.

Finally, the toxicity of the novel insulin composite crystals was tested in two different cell lines, namely Caco 2T and IEC18 cells (Figure 7A,B), which have an intestinal epithelial phenotype. Insulin composite crystals had no effect on lactate dehydrogenase (LDH) release, an index of cytotoxicity, up to a concentration of 100 μg/mL, and produced no discernible changes in cells by phase-contrast microscopy (Figure S5, Supporting Information). Thus, these crystals are nontoxic at the concentrations attained after subcutaneous injection.

## Conclusions

Insulin crystals grown in agarose and Fmoc-AA hydrogels have been successfully obtained in homogeneous batches compatible with *in vivo* subcutaneous administration, that is, samples containing a very narrow size distribution of small crystals (less than 10 μm diameter) free of any toxic additive. The physicochemical evaluation of both composite crystals has shown that these crystals are much more stable than insulin in solution, being pharmacologically active after keeping them at 50 °C during 7 days. In addition, insulin crystals grown in Fmoc-AA hydrogels showed an enhanced thermal stability, being stable up to 60 °C during 24 h. Significantly, this increase in stability also modifies the release profile of native insulin, turning it into a slow-release one, without altering the chemical structure of the protein. These results show that the nature of the hydrogel has a major impact on the physicochemical properties of the resulting crystals. Herein we have presented two novel thermally stable insulin formulations having different release profiles using the same native insulin. The capacity of tuning and improving the physicochemical properties of therapeutic proteins by simply crystallizing them in different hydrogels offers the possibility to expand the therapeutic window of novel biopharmaceutical formulations.

Insulin has been used in this work as a proof of concept; nevertheless, we are convinced that other therapeutic proteins can also be benefited from this approach. Thus, this work anticipates the potential advantages of using protein composite crystals for therapeutic purposes.

## Experimental Section

### Hydrogel Characterization

#### Transmission Electron Microscopy

Transmission electron microscopy of dried gels (xerogels) was conducted with a LIBRA 120 PLUS (Carl Zeiss). Hydrogels were vortexed and diluted twice with water. A drop of the fiber suspension obtained was placed on a 300-mesh copper grid and stained with uranyl acetate negative stain. The sample was dried at room temperature for 1h.

#### Circular Dichroism

The circular dichroism of Fmoc-AA hydrogels was recorded using the J-815 spectrophotometer of Jasco with a xenon lamp of 150 W. The mixtures were jellified into a 0.1 mm quartz cell (Hellma 0.1 mm quartz SuprasilR) using the protocols described below. Spectra were obtained from 200 to 350 nm with a 1 nm step and 0.5 s integration time per step at 25 °C. The data shown correspond to the average of 100 accumulations.

#### Rheological Characterization

For this, we used a Haake Mars III-controlled stress rheometer (Thermo Fisher Scientific, Waltham, MA), provided with a double cone-plate sensor of 60 mm diameter, 2° apex angle, 0.088 mm truncation, and made of titanium (sensor DC60/2° Ti L). In measurements, the gap between the double cone and the plate of the rheometer was equal to the truncation of the cone (0.088 mm), and a sample volume of 5.4 mL was required for this sensor. We measured the viscosity using a logarithmic ramp of shear rates. Separately, we obtained the storage (*G*′) and loss (*G*″) moduli of the samples by subjecting them to oscillatory shear strains of logarithmically increasing amplitude and a fixed frequency of 1 Hz. Three different samples were measured to ensure the statistical significance of the results. The mean values and standard deviations of each magnitude are provided in this work.

### Crystal’s Analysis

#### Crystallization Protocol

Human insulin, supplied by Sigma Aldrich (ref 11376497001 ROCHE), was dissolved in 20 mM HCl and the concentration was determined spectrophotometrically at 276 nm using an extinction coefficient (ε) of 1.04 mL/(mg cm). To obtain insulin crystals, we used the batch method by following a well-established sequential protocol consisting of the mix of the mother insulin solution (final concentration: 5 mg/mL), with ZnCl_2_ (final: 5.0 mM), sodium citrate, pH 7.0 (final: 22.0 mM) and water to a final volume of 200 μL in a 1.5 mL Eppendorf tube.^30^ To include the hydrogel, pre-warmed agarose solution at 0.5% (w/v) or Fmoc-AA dissolved in dimethyl sulfoxide (DMSO) (final concentration: 5% v/v) was added as the last step to produce the hydrogel at a final concentration of 0.05 and 0.2% w/v for agarose and Fmoc-AA, respectively. An identical protocol was used to assay the different gel concentrations initially tested. Samples were incubated at 20 °C for 24 h. For thermal stability evaluation, the samples were kept in an incubator set at 50 °C for a week or 24 h at 60 °C.

#### Dissolution Experiments

For the dissolution experiments, insulin crystals grown in both hydrogels were moved into the bulk solution with the help of a cat whisker and those crystals sticking to the wall were gently detached. Eppendorf tubes were then centrifuged at 11 000 rpm. The precipitant solution was replaced by PBS buffer, pH 7.4, prewarmed at 38 °C. The crystals were gently resuspended manually, and the tubes were shaken at 300 rpm and at 38 °C in a thermoshaker. Solution aliquots were taken at 2, 5, 10, 15, 20, 30, and 60 min. Tube samples were centrifuged at 11 000 rpm for 30 s, and 15 μL volume was extracted from the supernatant and diluted by adding 300 μL of water. Insulin in the solution was determined spectrophotometrically.

#### Differential Scanning Calorimetry

DSC experiments were carried out in a DASM-4 microcalorimeter equipped with capillary platinum cells at a scan rate of 2 °C/min. Crystal suspensions were readily diluted with crystallization buffer to a 1 mg/mL insulin concentration and immediately loaded into the calorimeter cell. The reference cell was loaded with the crystallization buffer. The samples were scanned up to 100 °C, cooled, and rescanned to check the reversibility of the thermograms. The experimental thermograms were corrected using a baseline obtained with both cells filled with crystallization buffer and normalized with the nominal insulin concentration. Then, the intrinsic heat capacity dependence was subtracted from the thermograms to give the excess heat capacity profiles.

#### HPLC Analysis

HPLC analyses were performed on an Agilent 1260 infinity. Analytical HPLC analyses were performed with an Agilent Poroshell 120 EC-C18, 2.7 μm, 50 mm × 4.6 mm column. Detection was by UV absorbance at 276 nm. The following eluents were used: (A) H_2_O + 0.1% trifluoroacetic acid (TFA); (B) MeCN + 0.1% TFA HPLC grade eluents, from 5 to 60% of MeCN in 8 min, and then from 60 to 95% of MeCN in 3 min, employed at a flow rate of 1 mL/min and filtered with a 0.2 μm filter prior to injection. The following HPLC method was used. The injection volume was 10 μL and detection wavelength was 276 nm. All experiments were conducted at 45 °C and all samples were filtered prior to injection. Crystal samples were diluted five times by adding 20 mM HCl and shaken at 1000 rpm for 45 min at 35 °C in a thermoshaker. Insulin solutions of known concentrations, 3, 8, 25, 75 and 225 μM, in 20 mM HCl were injected to obtain the calibration curve as a function of the total area of the peak (insulin peak) that absorbs at 276 nm and 10 min retention time. To quantify the insulin concentration in all HPLC experiments, the insulin peak total area was interpolated and a dilution factor of 1:5 was applied.

#### Mass Spectrometry

Mass spectra were recorded on a Bruker AutoFlex MALDI-ToF MS using a weight range from 1800 to 10 000 Da. Spectra were acquired for each sample in a positive ionization reflector mode (delay 270 ns, 19 kV accelerating voltage, variable laser intensity, typically more than 200 shots). Twenty microliters of sinapinic acid matrix (saturated solution of sinapinic acid in acetonitrile/0.1% TFA in water, 1:2) was mixed with 2 μL of sample. Two microliters of the resulting mixture was spotted onto the Bruker 384 stainless-steel MALDI-ToF plate for analysis in a Bruker MALDI-ToF Autoflex.

#### Scanning Electron Microscopy

Crystal samples were cross-linked by adding a commercial aqueous solution of glutaraldehyde 25% (v/v) (Sigma-Aldrich) to reach a final concentration of 5% (v/v). The mixture was homogenized with a pipette and allowed to cross-link by incubation at 20 °C for 24 h. The crystals were gently centrifugated, and the supernatant was removed and substituted by Milli-Q water. The process was repeated five times. Five microliters of the obtained aqueous cross-linked crystal suspension was manually extended on top of a carbon adhesive surface attached to an SEM pin stud and allowed to dry at room temperature overnight. The samples were then coated with a fine carbon layer and examined by SEM using a FEI Quanta 400 ESEM equipment. Crystal size distribution was measured from the SEM images using ImageJ 1.47 software. The final result was expressed as the average size of 100 crystals per sample. In each analyzed image, all crystals with at least one defined face were measured by taking the diagonal length of the biggest exposed face.

#### *In Vivo* Assays

All animal procedures in this study were carried out in accordance with the existing guidelines and were approved by the Animal Welfare Committee registry number CEEA 2011-354. Male Wistar rats of approximately 8 weeks obtained from Janvier Labs (Le Genest-Saint-Isle, France) were used. Animals were maintained at the Unit of Animal Research Biomedical Research Center, University of Granada, Granada, Spain, in specific pathogen-free conditions with free access to autoclaved tap water and food Harlan-Teklad 2014, Harlan Ibérica, Barcelona, Spain.

#### *In Vivo* Experiments

Rats were fasted overnight, and a sample for basal glycemia determination was obtained. Rats were then administered subcutaneously an insulin preparation or vehicle, namely, standard human insulin, agarose, and Fmoc-AA crystals, or the respective vehicle. The samples were subjected to 30 min of UV radiation for sterilization purposes. Insulin preparations were subjected to incubation at 20 or 50 °C for 1 week and 60 °C for 24 h in order to test the thermostability. Blood samples were obtained at 0.5, 1, 2, 4, 6, and 8 h after administration of Fmoc-AA crystals, or at 20, 40, 60, 90, 120, and 180 min after administration of agarose crystals, owing to the different time response (*n* = 10 except *n* = 7 for native insulin with thermal stress and *n* = 4 for the groups receiving gel without insulin). Blood samples were drawn from a little incision of the tip of the rat tails and the blood was spun; the plasma was stored at −80 °C until assayed for glucose levels.

#### Measurement of Glucose Levels

Glucose plasma levels were measured using the enzymatic kit from SpinReact (ref 1001192) following the manufacturer’s instructions.

#### *In Vivo* Data and Statistical Analysis

Samples were run at least in triplicate and the results are expressed as mean ± standard error of the mean (SEM). Differences among means were tested for statistical significance by two-way analysis of variance (ANOVA) and a posteriori Fisher’s least significant difference tests on preselected pairs. All analyses were carried out with the GraphPad Prism 6 (La Jolla, CA). Differences were considered significant at *P* < 0.05.

#### Toxicological Studies

IEC18 (passages 56–61) and Caco 2T cells (passages 65–70) were cultured in standard conditions to confluence in 96-well plates, then exposed to the composite crystals for 16 h in fresh culture medium (Dulbecco’s modified Eagle’s medium with 10% fetal bovine serum, 100 IU/mL penicillin, 0.1 mg/mL streptomycin, 25 μg/mL Fungizone (Gibco), and 2 mM l-glutamine, Sigma). LDH activity was measured in the supernatant using Pierce cytotoxicity kits (ref 88954), while cell appearance was documented with an Olympus CKX41 microscope.

## Supporting Information Available

SEM images of all insulin crystals samples (Figure S1); HPLC calibration curve (Figure S2); HPLC analysis of control crystals (Figure S3); mass spectra of all samples (Figure S4); contrast phase microscopy of the two cell lines used to study the cytotoxicity (Figure S5) (PDF)
