# Supplementary material for: Insulin Crystals Grown in Short-Peptide Supramolecular Hydrogels Show Enhanced Thermal Stability and Slower Release Profile
Source: ACS Appl Mater Interfaces. 2021 Mar 4;13(10):11672–82. doi: 10.1021/acsami.1c00639 (PMC8479728; doi:10.1021/acsami.1c00639)
Supplement: Supplementary file 1 — am1c00639_si_001.pdf [file am1c00639_si_001.pdf]

Supporting Information

Insulin crystals grown in short peptide supramolecular hydrogels show enhanced thermal stability and slower release profile

Rafael Contreras-Montoya, María Arredondo-Amador, Guillermo Escolano-Casado, Mari C. Mañas-Torres, Mercedes González, Mayte Conejero-Muriel, Vaibhav Bhatia, Juan J. Díaz-Mochón, Olga Martínez-Augustin, Fermín Sánchez de Medina, Modesto T. Lopez-Lopez, Francisco Conejero-Lara, José A. Gavira and Luis Álvarez de Cienfuegos**

Corresponding authors:

L. Álvarez de Cienfuegos. Departamento de Química Orgánica, Universidad de Granada, (UGR), C. U. Fuentenueva, Avda. Severo Ochoa s/n, E-18071 Granada; Instituto de Investigación Biosanitaria ibs.GRANADA, Spain. Email: lac@ugr.es

J. A. Gavira. Laboratorio de Estudios Cristalográficos, Instituto Andaluz de Ciencias de la Tierra (Consejo Superior de Investigaciones Científicas-UGR), Avenida de las Palmeras 4, 18100 Armilla, Granada, Spain. Email: jgavira@iact.ugr-csic.es

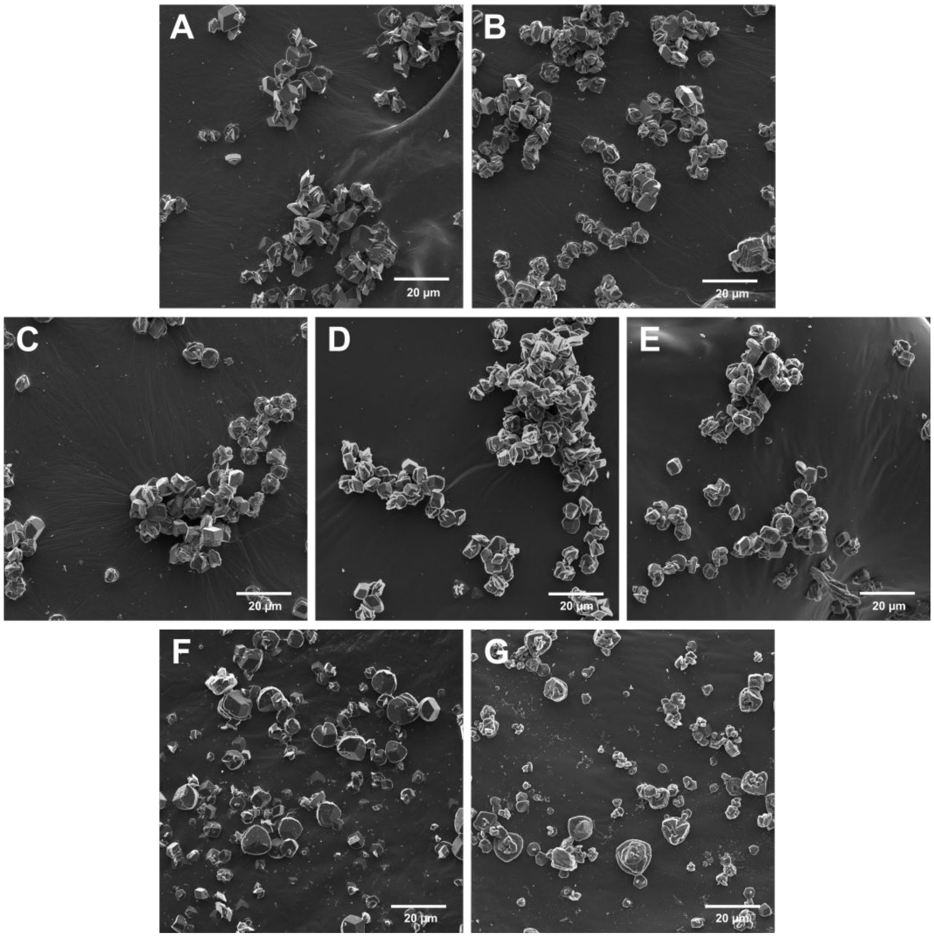

Figure S1. SEM images of insulin crystals: A) Control crystals at RT; B) Control crystals kept at 50ºC during 7 days; C) Fmoc-AA crystals kept at RT; D) Fmoc-AA crystals kept at 50 ºC during 7 days; E) Fmoc-AA crystals kept at 60 ºC during 24 hours; F) Agarose crystals kept at RT; G) Agarose crystals kept at 50 ºC during 7 days.

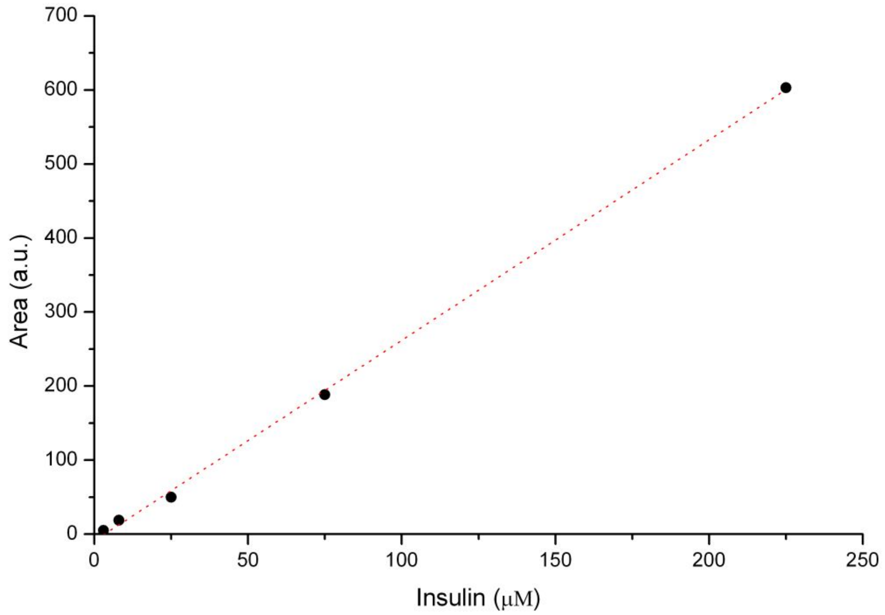

Figure S2. Calibration curve of insulin concentrations measured at 276 nm.

A)

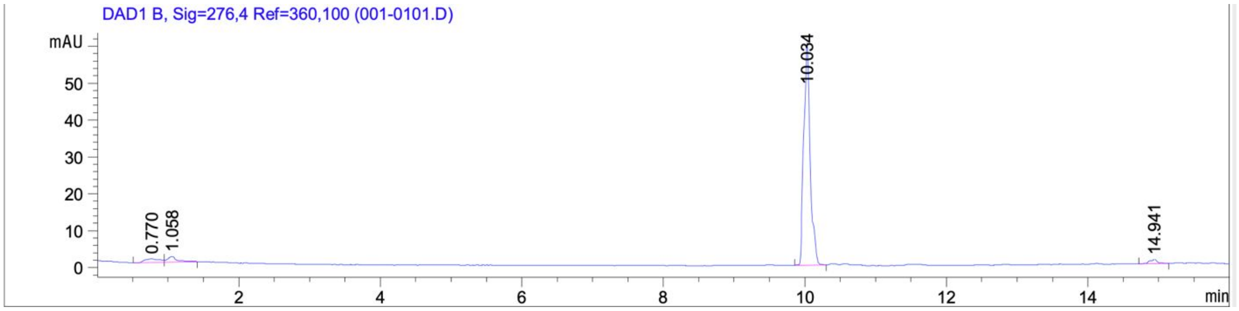

B)

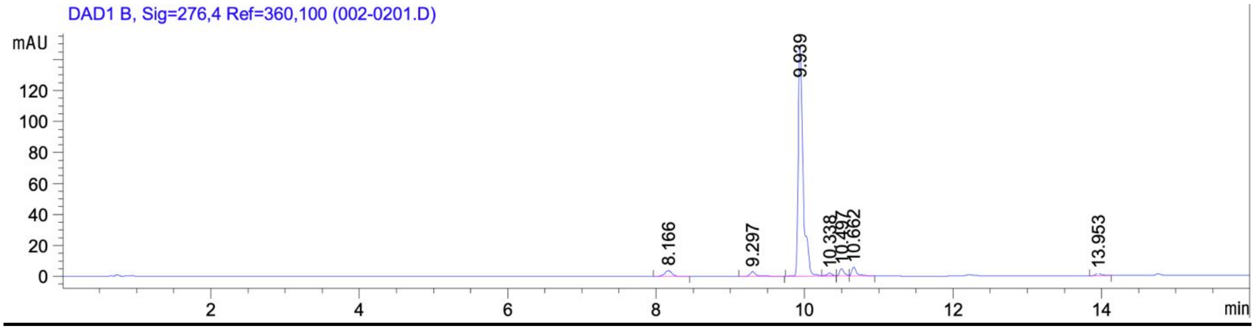

Figure S3. HPLC analysis of control crystals at RT (A) and 50 °C (B).

Insulin control

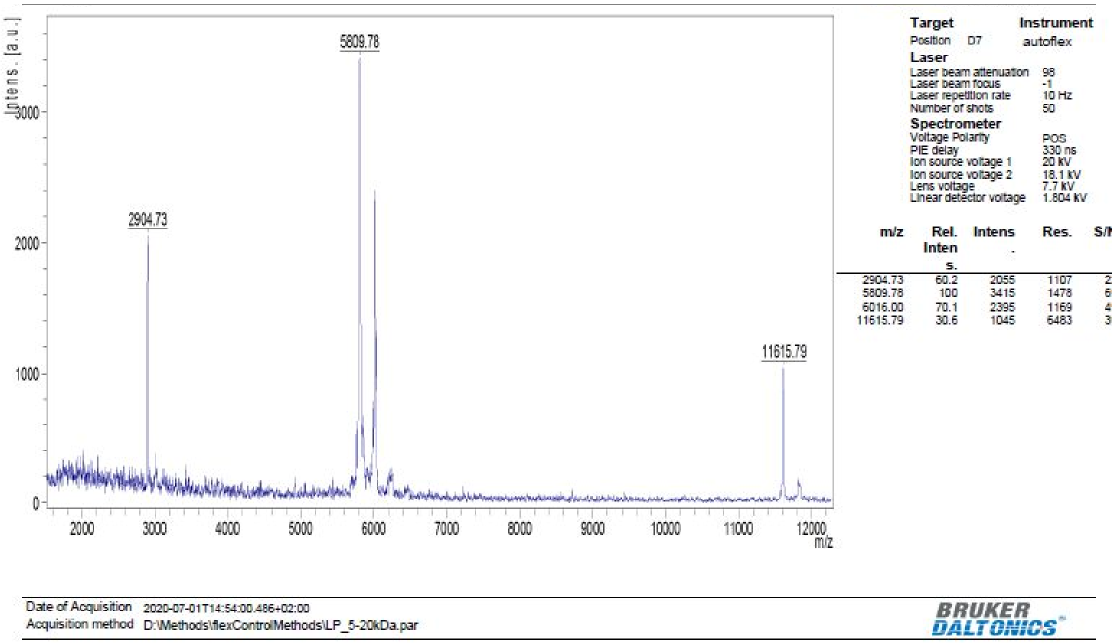

$(M+2H)^{+2}$ = 2,904; $(M+1H)^{+1}$ = 5,808 and $(2M+1H)^{+1}$ = 11,615 – impurity = 6,016. This impurity, that appears in most of the samples, might be assigned to the byproduct formed by the reaction of insulin monomer with the matrix adduct sinapinic acid (3,5-dimethoxy-4-hydroxycinammic acid). This type of reaction has been previously observed using the same Mass spectrometry technique, see C. K. Fagerquist et al. *J. Am. Soc. Mass Spectrom*. 2012, **23**, 2102-2114.

Control crystals

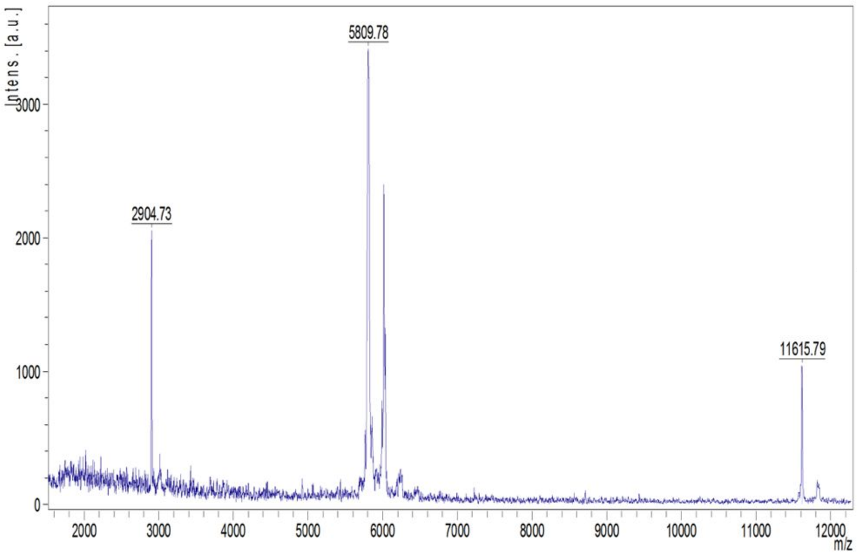

Target
Position D7

Instrument
autoflex

Laser
Laser beam attenuation 98
Laser beam focus -1
Laser repetition rate 10 Hz
Number of shots 50

Spectrometer
Voltage Polarity POS
PIE delay 330 ns
Ion source voltage 1 20 kV
Ion source voltage 2 18.1 kV
Lens voltage 7.7 kV
Linear detector voltage 1.804 kV

m/z	Rel. Intens.	Intens.	Res.	S/N
2904.73	60.2	2055	1107	22
5809.78	100	3415	1478	69
6016.00	70.1	2395	1169	49
11615.79	30.6	1045	6483	39

Date of Acquisition 2020-07-01T14:54:00.486+02:00
Acquisition method D:\Methods\flexControlMethods\LP_5-20kDa.par

BRUKER DALTONICS

Control crystals 50°C

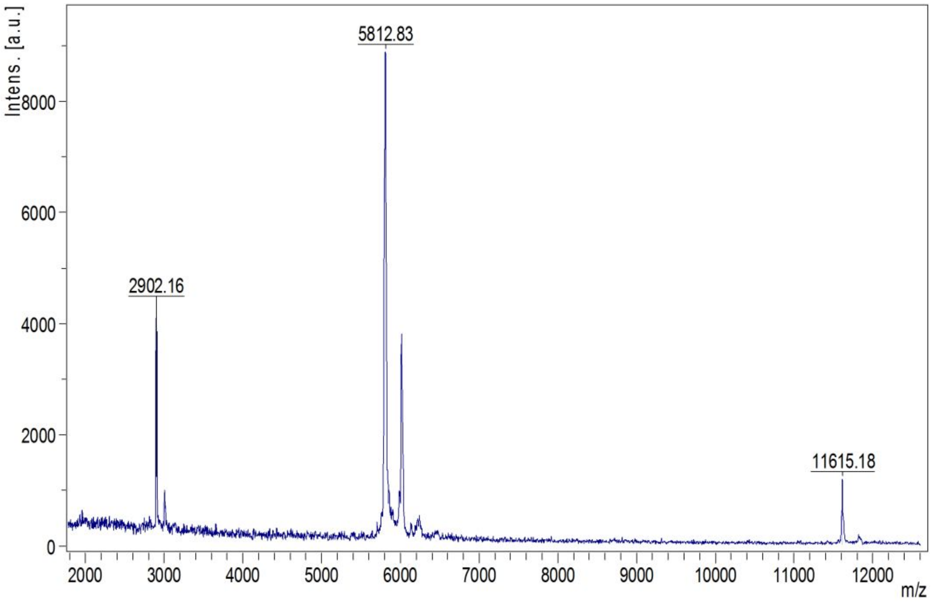

Target
Position P6

Instrument
autoflex

Laser
Laser beam attenuation 55
Laser beam focus -1
Laser repetition rate 10 Hz
Number of shots 100

Spectrometer
Voltage Polarity POS
PIE delay 330 ns
Ion source voltage 1 20 kV
Ion source voltage 2 18.1 kV
Lens voltage 7.7 kV
Linear detector voltage 1.804 kV

m/z	Rel. Intens.	Intens.	Res.	S/N
2896.51	12.9	1142	819	10
2902.16	13.4	1188	734	13
5812.83	100	8890	614	105
6014.11	42.3	3760	5036	45
11615.18	13.5	1200	3151	27

Date of Acquisition 2020-06-24T12:27:07.729+02:00
Acquisition method D:\Methods\flexControlMethods\LP_5-20kDa.par

BRUKER DALTONICS

Fmoc-AA crystals RT

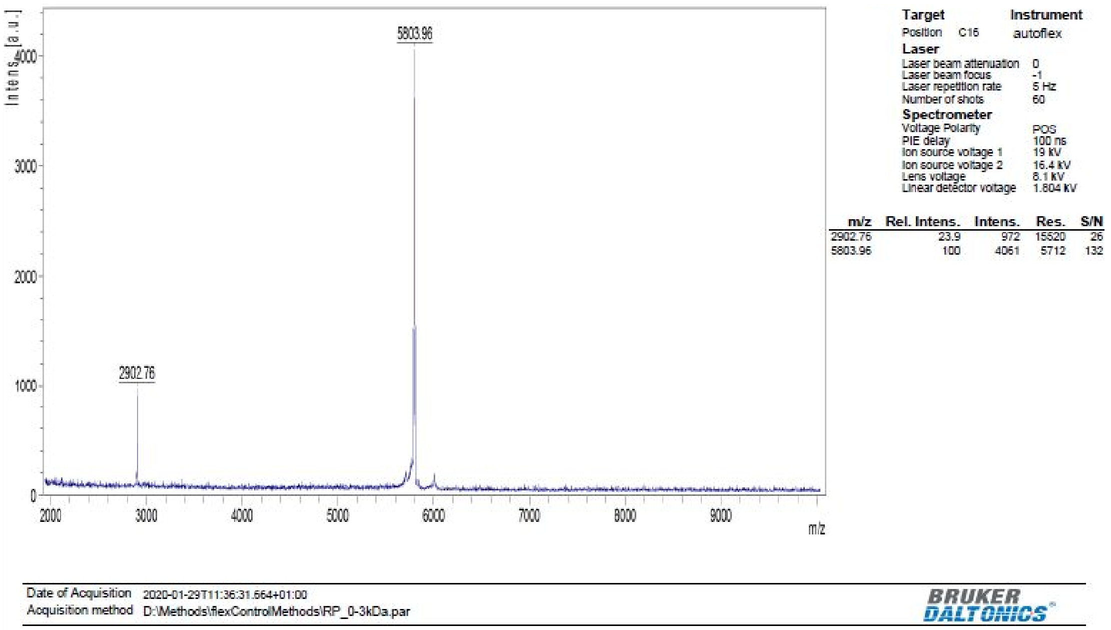

m/z	Rel. Intens.	Intens.	Res.	S/N
2902.76	23.9	972	15520	26
5803.96	100	4061	5712	132

Fmoc-AA crystals 50°C

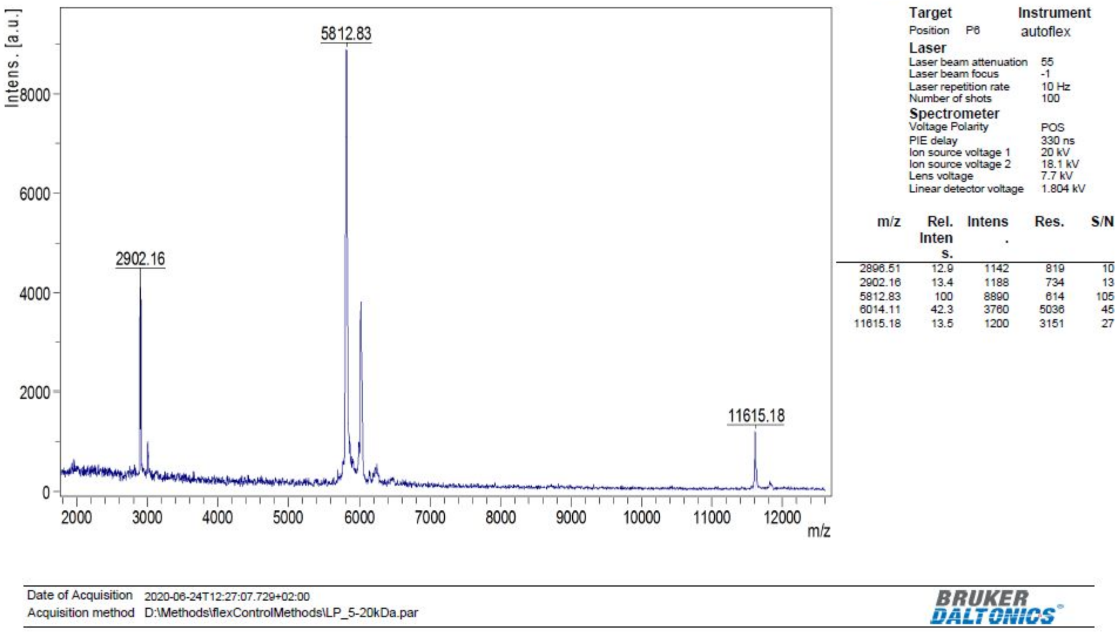

m/z	Rel. Intens.	Intens.	Res.	S/N
2896.51	12.9	1142	819	10
2902.16	13.4	1188	734	13
5812.83	100	8890	614	105
6014.11	42.3	3760	5036	45
11615.18	13.5	1200	3151	27

Fmoc-AA crystals 60°C

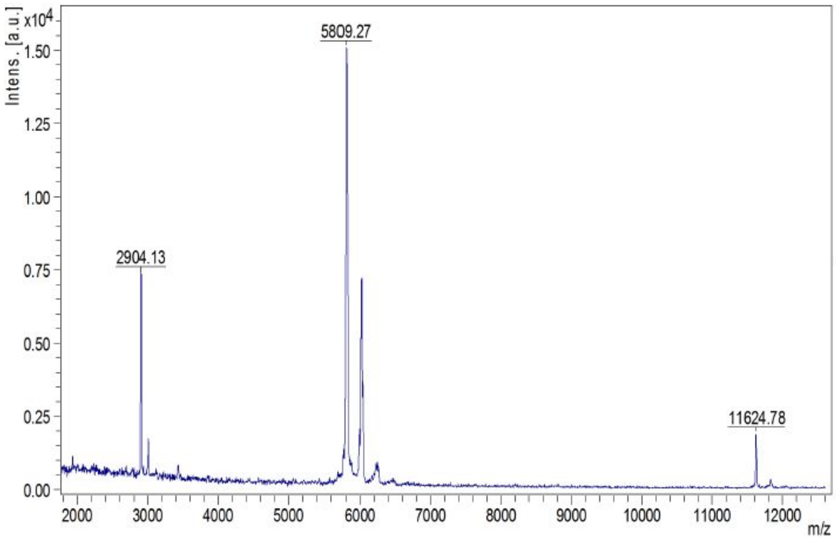

Target
Position L6

Instrument
autoflex

Laser

Laser beam attenuation	55
Laser beam focus	-1
Laser repetition rate	10 Hz
Number of shots	100

Spectrometer

Voltage Polarity	POS
PIE delay	330 ns
Ion source voltage 1	20 kV
Ion source voltage 2	18.1 kV
Lens voltage	7.7 kV
Linear detector voltage	1.804 kV

m/z	Rel. Intens.	Intens.	Res.	S/N
2904.13	48.4	7290	6611	39
5798.02	62.0	9345	1540	97
5809.27	100	15075	1589	158
6019.69	45.6	6880	3415	73
11624.78	12.6	1895	1180	39

Date of Acquisition 2020-06-24T12:41:00.290+02:00
Acquisition method D:\Methods\flexControlMethods\LP_5-20kDa.par

BRUKER DALTONICS

Agarose crystals RT

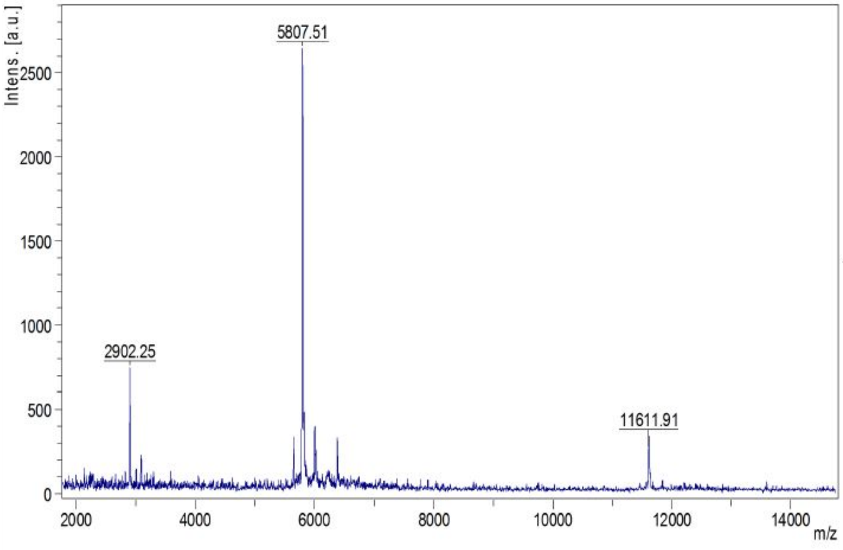

Target
Position O3

Instrument
autoflex

Laser

Laser beam attenuation	100
Laser beam focus	-1
Laser repetition rate	10 Hz
Number of shots	50

Spectrometer

Voltage Polarity	POS
PIE delay	330 ns
Ion source voltage 1	20 kV
Ion source voltage 2	18.1 kV
Lens voltage	7.7 kV
Linear detector voltage	1.804 kV

m/z	Rel. Intens.	Intens.	Res.	S/N
2902.25	28.2	745	2875	2[illegible]
5807.51	100	2645	1997	7[illegible]
6009.91	13.8	365	5934	1[illegible]
11611.91	9.83	260	7247	1[illegible]

Date of Acquisition 2020-09-10T13:30:25.942+02:00
Acquisition method D:\Methods\flexControlMethods\LP_5-20kDa.par

BRUKER DALTONICS

Agarose crystals 50 °C

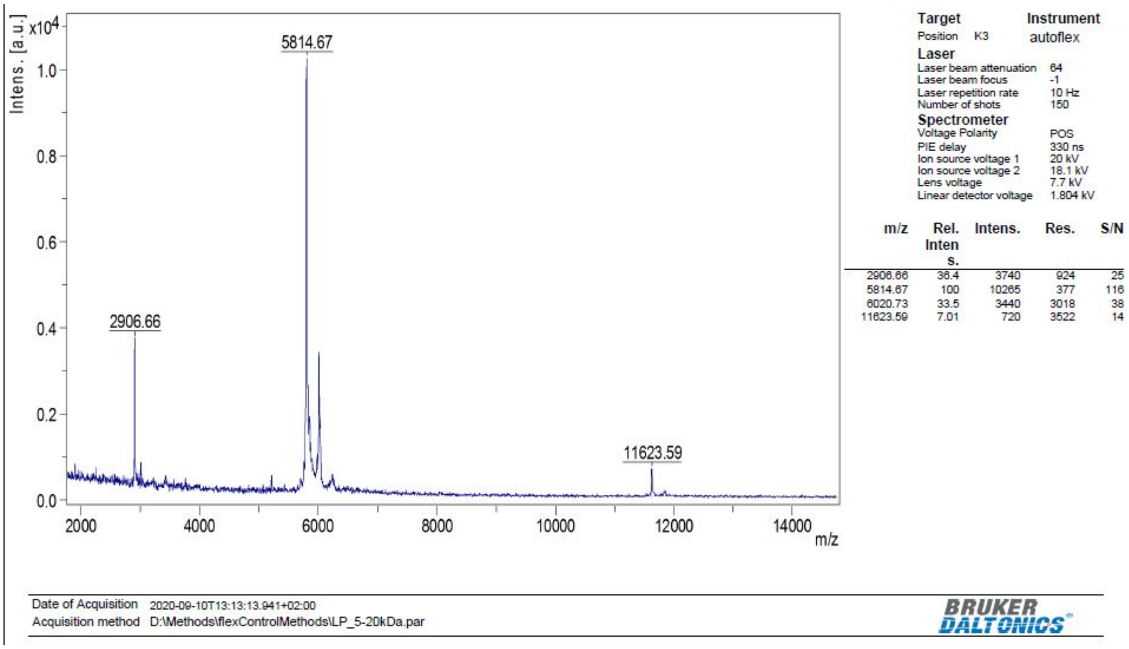

m/z	Rel. Intens.	Intens.	Res.	S/N
2906.66	36.4	3740	924	25
5814.67	100	10265	377	116
6020.73	33.5	3440	3018	38
11623.59	7.01	720	3522	14

Figure S4. Mass spectra of insulin samples.

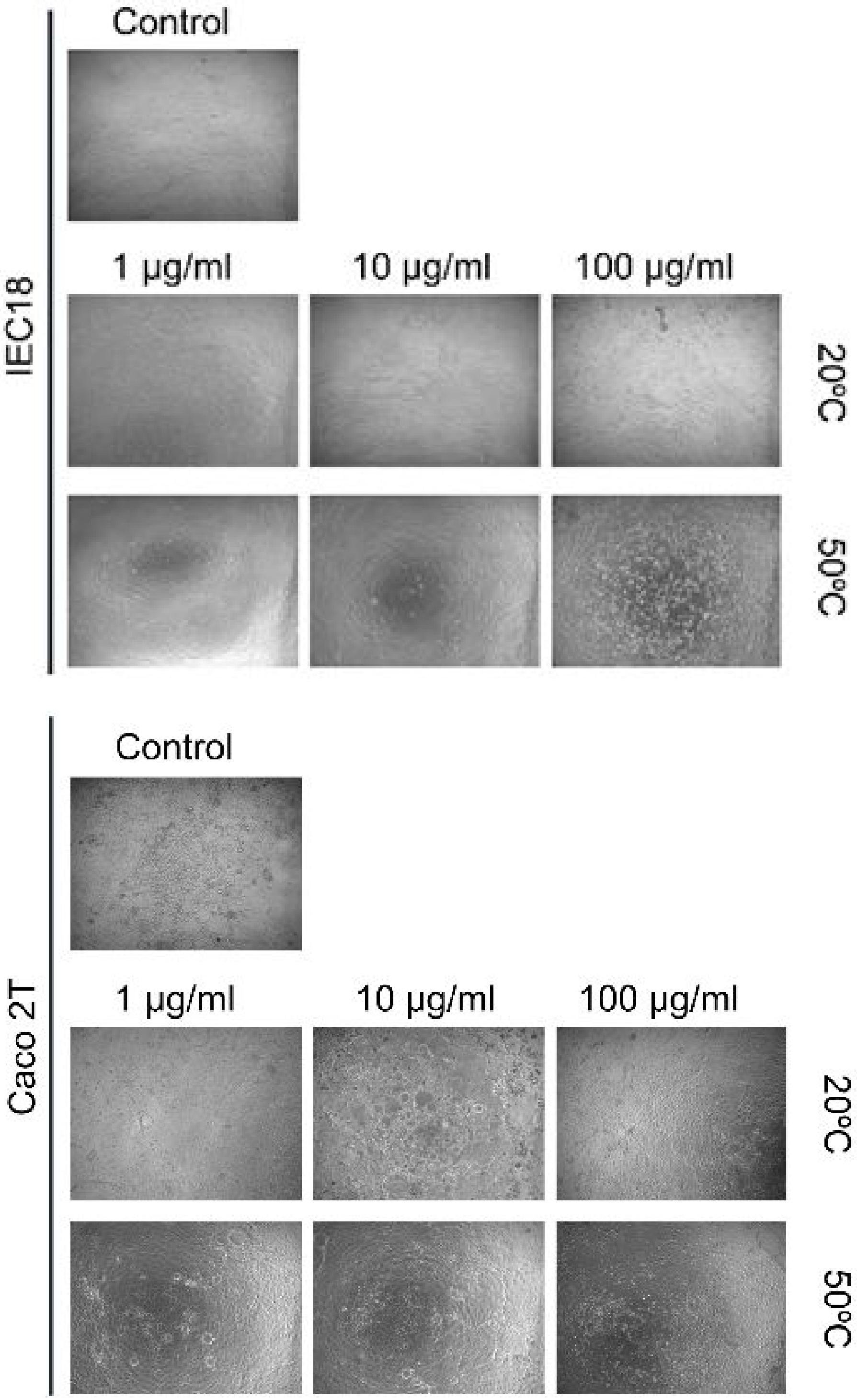

Figure S5. Contrast phase microscopy of the two cell lines used to study the cytotoxicity.
